# A rare presentation of metastatic prostate cancer, initially a suspect for urothelial cell carcinoma of the ureter: a case report

**DOI:** 10.1186/s12894-017-0227-1

**Published:** 2017-05-26

**Authors:** Ho Seok Chung, Myung Soo Kim, Yang Hyun Cho, Eu Chang Hwang, Seung Il Jung, Taek Won Kang, Dong Deuk Kwon, Suk Hee Heo, Chan Choi

**Affiliations:** 1 Department of Urology, Chonnam National University Medical School, 42 Jebongro, Donggu, Gwangju, 501-757 Republic of Korea; 2 Department of Radiology, Chonnam National University Medical School, Gwangju, Republic of Korea; 3 Department of Pathology, Chonnam National University Medical School, Gwangju, Republic of Korea

**Keywords:** Neoplasm metastasis, Prostate cancer, Ureter

## Abstract

**Background:**

The most common metastatic sites of prostate cancer are the lymph nodes and bone. Ureteral metastasis from prostate cancer is very unusual and only a few cases have been reported.

**Case presentation:**

We describe a 76-year-old male with ureteral metastasis of prostate cancer along with a review of the literature. Initially, based on the diagnostic evaluation, urothelial cell carcinoma of the left distal ureter was suspected. Nephroureterectomy with bladder cuff excision was performed. The final pathologic diagnosis was prostate cancer metastatic to the ureter.

**Conclusion:**

Although rare and the mechanistic link between prostate cancer and distant ureteral metastasis has not been clarified on a clinical basis, this would be included in the differential diagnosis of ureteral lesions in patients with a history of prostate cancer. It is important to recognize this unusual manifestation so that timely appropriate treatment can be initiated.

## Background

Prostate cancer, one of the most common malignancies in aging men, commonly spreads to lymph nodes and bone [1]. Ureteral metastasis from other primary cancers is very rare, and prostate cancer metastatic to the ureter is extremely rare, as only 45 cases have been reported worldwide in the last century [2, 3]. Herein, we describe a patient with hydronephrosis secondary to a ureteral tumor caused by metastasis from prostate cancer.

## Case presentation

A 76-year-old male visited the emergency room in June 2014 because of left flank pain. His past medical history was significant for advanced prostate cancer treated with androgen deprivation therapy (ADT). According to medical records, he first presented at our outpatient department with urinary obstructive symptoms and was diagnosed with prostate cancer (clinical stage T3bN0M0), with an initial serum prostate specific antigen (PSA) level of 80.69 ng/ml 2 years earlier. At that time, we recommended ADT plus radiation for the treatment of the prostate cancer. However, the patient only received ADT. After 9 months of complete androgen blockade therapy, the PSA had decreased to 0.39 ng/ml, but the patient was lost to follow-up and treatment.

**Fig. 1:**
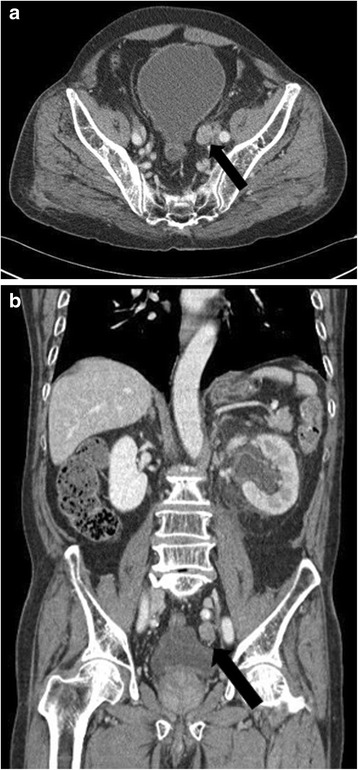
Abdominal computed tomography showing a left ureteral mass with hydronephrosis. **a** axial view, **b** coronal view

**Fig. 2:**
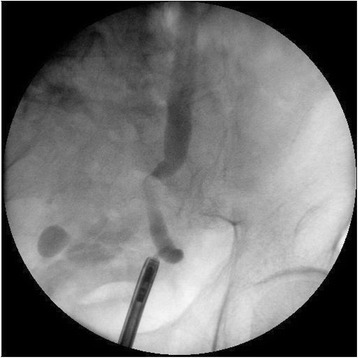
Retrograde pyelography, showing smooth marginated filling defects in the left distal ureter

When he again presented at the emergency room in June 2014, the PSA level was 6.75 ng/ml. Abdominal computed tomography (CT) revealed a left distal ureteral enhancing mass about 2.1 cm in length causing hydronephrosis, and no lymphadenopathy (Fig. 1). We initially performed left percutaneous nephrostomy for symptomatic hydronephrosis. Retrograde pyelography showed smooth, marginated filling defects in the left distal ureter (Fig. 2). Cytology showed no pathological results.

**Fig. 3:**
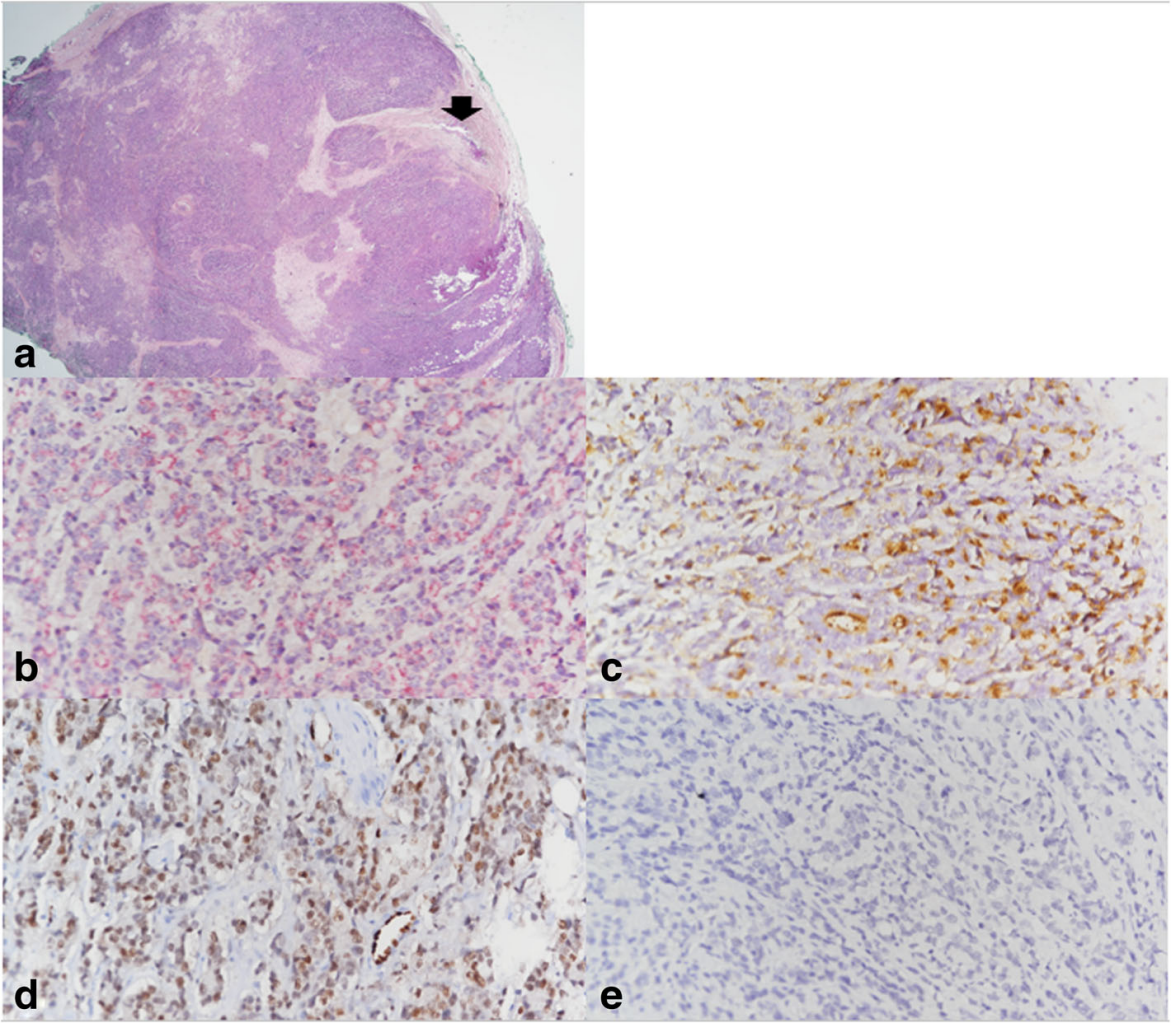
Pathological features of the involved ureter. **a** Solid sheet of hyperchromatic cells are noted around the ureter. Arrow indicates ureter. (hematoxylin-eosin staining, ×10) (**b**, **c**, **d**, **e**) The tumor cells were positive for p504S, prostate specific antigen (PSA), and ERG, and negative for p63 (immunohistochemical stain, ×200)

Because of suspected urothelial cell carcinoma of the left distal ureter, nephroureterectomy with bladder cuff excision was performed. Pathological examination revealed a lesion consisting of hyperchromatic cells around the ureter (Fig. 3a). Immunohistochemical staining was strongly positive for prostate cancer markers, including p504S, PSA, and ERG, and negative for p63 (Fig. 3b-e). These findings confirmed a diagnosis of prostate carcinoma metastatic to the left ureter, with no evidence of urothelial cell carcinoma. The tumor invaded the adventitia and muscularis of the ureter, but the distal ureteral surgical margin was not involved by tumor cells.

After the operation, the patient was treated with complete androgen blockade therapy. However, at the 3-month follow-up, the PSA level increased to 8.73 ng/ml. At the 1-year follow up, further progression with multiple bone metastases, metastatic lymphadenopathy, and right ureteral metastasis led to docetaxel chemotherapy following enzalutamide therapy, but terminating in death after the year.

## Discussion

There is increasing discussion about the risk of development of a second primary cancer in prostate cancer patients [4]. Braisch et al. reported an increased risk of a subsequent primary cancer in the renal pelvis and ureter [5]. Ureteral lesions can also occur by metastasis from primary cancer. The most common malignancies that metastasize to the ureter are breast cancer, gastric cancer, and colorectal cancer [6]. However, ureteral metastasis from any type of primary cancer is unusual, because the ureters have segmental lymphatic circulation without continuation in the ureteral wall. Moreover, ureteral metastasis from prostate cancer is extremely rare, because there is no direct periureteral sheath drainage from the prostate [7]. The ureters can be affected by prostate cancer causing hydronephrosis through direct invasion of the tumor around the intravesical ureter. Prostate cancer may metastasize to the ureter through dissemination of malignant cells to the retroperitoneal lymph nodes near the ureter, via the periureteral lymphatic pathway [8].

A total of 38 cases of ureteral metastases from prostate cancer were described by Haddad in 1999 [2]. Since then, few cases have been reported [3, 6]. In these cases, the most common symptom was flank pain caused by ureteral obstruction, as in our case. In addition, most ureteral metastases were treated by nephroureterectomy because of presumed upper urothelial carcinoma [3]. However, before surgery, diagnostic ureteroscopy and biopsy would be reasonable options for the differential diagnosis [9]. Because nephroureterectomy might have been avoided, and the ureteral mass could be regressed under antiandrogen treatment. For severe flank pain with hydronephrosis, immediate percutaneous nephrostomy or double J stent might be a good choice. Gross hematuria is rarely observed, possibly because most ureteral metastasis occurs beneath the mucosa and by invasion from surrounding tissues [6]. Most case series reported that primary prostate cancer metastatic to ureter had a Gleason score (GS) ≥ 7 [3]. In our case, transrectal ultrasound (TRUS)-guided biopsy revealed prostate cancer with GS 9 (4 + 5). It is possible that prostate cancer with a high GS is associated with the risk of ureteral metastasis [3].

## Conclusion

Although rare, the urologist should consider metastatic disease in the differential diagnosis of ureteral lesions in a patient with a history of prostate cancer with a high GS. If ureteral metastasis is confirmed by ureteroscopic biopsy before definitive treatment such as nephroureterectomy, segmental ureterectomy and ureteroureterostomy could be applied in this condition for preservation of ipsilateral kidney. In addition, conservative treatment using nephrostomy or double J stenting may be helpful to relieve urinary obstructive symptoms.

## Abbreviation

ADT: Androgen deprivation therapy
CT: Computed tomography
GS: Gleason score
PSA: Prostate specific antigen
TRUS: Transrectal ultrasound
